# African Swine Fever in Smallholder Sardinian Farms: Last 10 Years of Network Transmission Reconstruction and Analysis

**DOI:** 10.3389/fvets.2021.692448

**Published:** 2021-07-30

**Authors:** Sandro Rolesu, Daniela Mandas, Federica Loi, Annalisa Oggiano, Silvia Dei Giudici, Giulia Franzoni, Vittorio Guberti, Stefano Cappai

**Affiliations:** ^1^Sardinian Regional Veterinary Epidemiological Observatory, Istituto Zooprofilattico Sperimentale della Sardegna “G. Pegreffi”, Cagliari, Italy; ^2^Department of Animal Health, Istituto Zooprofilattico Sperimentale Della Sardegna, Sassari, Italy; ^3^ISPRA—Institute for Environmental Protection and Research, Rome, Italy

**Keywords:** African swine fever, smallholder farms, traditional pig farming system, outdoor pig farm, biosecurity measure, secondary case, kernel function, mathematical model

## Abstract

African swine fever (ASF) is a viral disease of suids that frequently leads to death. There are neither licensed vaccines nor treatments available, and even though humans are not susceptible to the disease, the serious socio-economic consequences associated with ASF have made it one of the most serious animal diseases of the last century. In this context, prevention and early detection play a key role in controlling the disease and avoiding losses in the pig value chain. Target biosecurity measures are a strong strategy against ASF virus (ASFV) incursions in farms nowadays, but to be efficient, these measures must be well-defined and easy to implement, both in commercial holdings and in the backyard sector. Furthermore, the backyard sector is of great importance in low-income settings, mainly for social and cultural practices that are highly specific to certain areas and communities. These contexts need to be addressed when authorities decide upon the provisions that should be applied in the case of infection or decide to combine them with strict preventive measures to mitigate the risk of virus spread. The need for a deeper understanding of the smallholder context is essential to prevent ASFV incursion and spread. Precise indications for pig breeding and risk estimation for ASFV introduction, spread and maintenance, taking into account the fact that these recommendations would be inapplicable in some contexts, are the keys for efficient target control measures. The aim of this work is to describe the 305 outbreaks that occurred in domestic pigs in Sardinia during the last epidemic season (2010–2018) in depth, providing essential features associated with intensive and backyard farms where the outbreaks occurred. In addition, the study estimates the average of secondary cases by kernel transmission network. Considering the current absence of ASF outbreaks in domestic pig farms in Sardinia since 2018, this work is a valid tool to specifically estimate the risk associated with different farm types and update our knowledge in this area.

## Introduction

African swine fever (ASF) is a devastating disease of domestic and wild pigs, frequently resulting in the death of infected animals. The disease causes significant losses in the pig sector due to its transcontinental spread and the lack of a licensed vaccine or treatment; thus, it currently represents one of the most important infectious and lethal swine diseases (1). The aetiological agent is the ASF virus (ASFV), a large double-stranded DNA virus that mainly infects myeloid cells, such as monocytes, macrophages, and dendritic cells (2, 3). Currently, 24 ASFV genotypes have been identified based on a fragment of the variable region of the B646L gene, which encode for the major protein p72 (3). So far, only genotype I ASFV isolates have been reported in Sardinia (4–6). Specific ASFV antibodies appear 7–10 days post-infection (dpi) (7), while clinical symptoms strictly depend on the different forms of the disease (8). The most common symptoms are fever, loss of appetite, lack of energy, abortion and hemorrhage (9). Sudden death may occur. Virulent ASFV isolates are generally fatal (death occurs within 10 days), while animals infected with attenuated ASFV strains may not show typical clinical signs (8). Even though the human population is not susceptible to the disease, ASF represents a global threat, given the associated considerable sanitary and socio-economic consequences. In fact, to date, ASF is widespread in over 30% of European, Asian and African countries, with a total of 8,551 ongoing outbreaks worldwide and Europe accounting for 67% of those reported outbreaks (10). Greater concerns are associated with the spread of the disease in China, which retains the largest pig production market (11, 12). There is a lack of information about ASF epidemiology, particularly in relation to different farm types. Despite the fact that the overall spread has been quantified (13, 14), ASFV's capacity for transmission between different farm types has never been defined. Furthermore, different basic reproduction number estimations have been provided for both wild and bred pig populations (4, 15–25), but are limited to a specific epidemic period and do not allow for a comparison to be made between different farm types. Despite the fact that the main risk factors are well-known overall, they are not specifically tailored to commercial or backyard farms (26). Several problems arise from the definition of secondary ASF cases. In fact, even though the ASF Diagnostic Manual (27) includes the condition “secondary cases epidemiologically correlated to primary case” for an outbreak declaration, the lack of a specific epidemiological correlation definition in terms of space and time makes this condition difficult to apply. The ASF risk estimation is even more complicated when considering the three different types of European pig farms described by the Working Document of the Directorate General for Health and Food Safety: “African swine fever strategy for Eastern part of the European Union” (SANTE/7113/2015-Rev 12). In this document, pig farms are classified as non-commercial, commercial, and outdoor farms. As underlined by Bellini et al. (28), this classification takes into account the commercial attitude of the holdings rather than the size of the farm or the type of establishment, thus making the application of most established biosecurity measures difficult (28–30). Furthermore, the last EFSA opinion on ASF and outdoor farming system underlines the lack of specific and harmonized system to categorize different types of pig farms (31). Considering the limited number of studies available on smallholder pig farms, in-depth evaluation of field data is required to define ASF risk factors specific to these types of farms, evaluate target biosecurity measures, and estimate the efficiency of these measures in European countries (32–36). Otherwise, finding the right context for such a specific, in-depth study could be difficult for several reasons. In fact, a robust epidemiological evaluation is more complete and detailed if the epidemic is halted or if there is epidemiological silence for at least 1 year (37). In addition, backyard farms are not common in all countries or not present in all forms (i.e., indoor, and outdoor). Even though Sardinia has not yet been declared free from ASF, the island context seems to be appropriate for the purpose of this work, given that the last ASFV outbreak in domestic pigs dates back to 2018 and the last virus finding in the wild boar to April 2019 (38). This allows to provide in details the risk factors associated with the occurrence of ASF at farm level. The aim of this work is to provide a descriptive analysis of the Sardinian farms where ASF outbreaks occurred during the past 10 years. Details on the farm type, biosecurity measures applied, ASFV laboratory results and clinical signs are included. Furthermore, this study aims to estimate the most likelihood ASF transmission network applying nearest-neighbor and uniform kernel function and compare these networks with that one described by official veterinarian reports. Finally, multilevel logistic mixed models were applied to establish the main farm' characteristics involved in the probability of observing an untimely outbreak.

## Materials and Methods

### The Epidemiology of ASF in Sardinia

**Figure 1:**
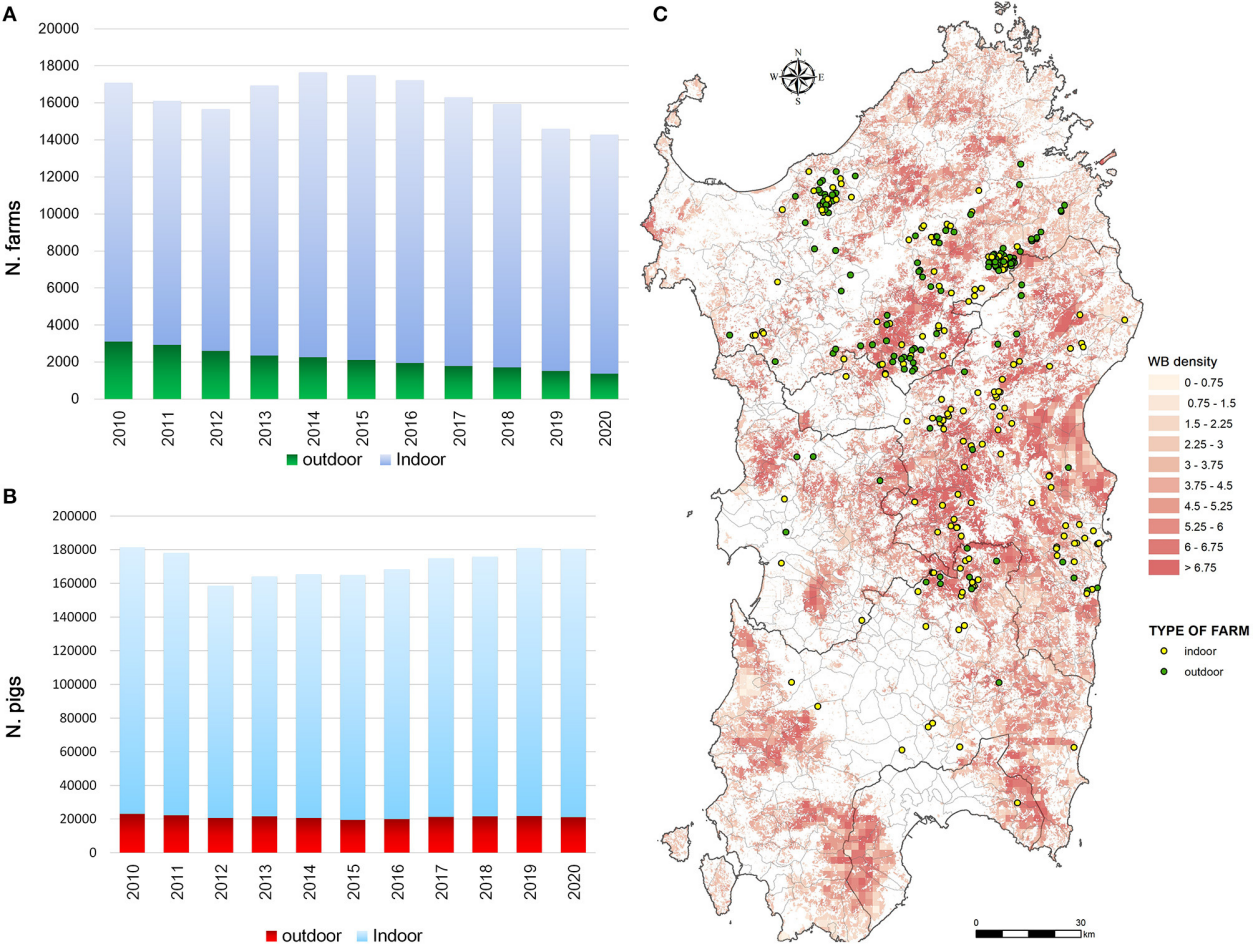
Sardinian epidemiological context. **(A)** Total number of farms in Sardinia and **(B)** the number of domestic pigs bred in domestic pig farms by years, from 2010 to 2020. Data are collected by the Italian National Database and correspond to the annual animal census (30th June). **(C)** Outbreaks occurred in indoor and outdoor domestic pig farms from 2010 to 2018 over the wild boar density by km2.

**Figure 2:**
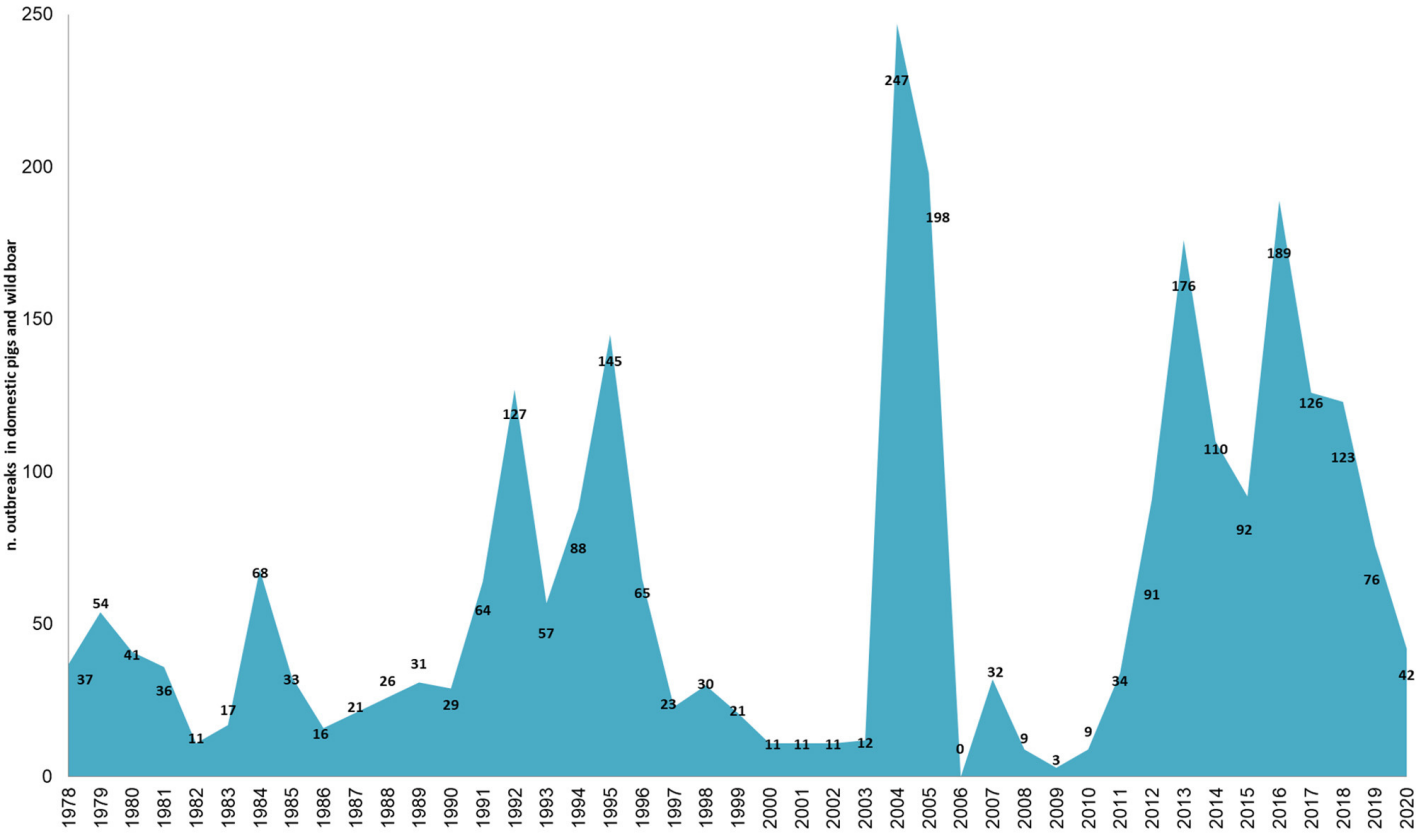
Number of African swine fever cases (i.e., outbreaks in domestic pig farms or in illegal free-ranging pigs, and cases in wild boar) reported in Sardinia from 1978 (by suspicion data on SIMAN) to 2020. The last notified outbreak in domestic pig farms occurred in September 2018. From April 2019, ASF outbreaks are limited to seropositive wild boar.

Sardinia has been affected by ASFV since 1978 and presents a particular ASF epidemiological context that is worth describing. While the rest of Europe is infected by ASFV genotype II, the island of Sardinia is the only part of the continent where ASFV genotype I has spread (4–6). Sardinia is the only area where ASFV has infected three porcine populations (i.e., domestic pigs, wild boar, and illegal free-ranging pigs) (6, 39, 40). As described by Wilkinson (41), free-ranging pig breeding has been a fundamental part of the agropastoral Sardinian culture for several decades. Despite the fact that free-range pig keeping is illegal in Sardinia, it was largely practiced until a few years ago, when several culling actions have been taken to reduce this population (39). During these actions, several ASFV-positive animals were detected (6, 39). In Sardinia, swine husbandry has been a secondary activity compared to sheep livestock production (40–43). Thus, domestic pig farms have a familiar or working relationship with other farms or are mainly for self-consumption, and only 5% are commercial farms (44). Furthermore, over about 16,000 total farms officially recorded in National Italian Database (BDN), the proportion of indoor farms was significantly higher (75–80%) than outdoor farms (20–25%) (Figure 1A), as well as the total number of domestic pig bred (Figure 1B). The number of pigs bred in indoor farms remained constant all over the years, while this increased in outdoor farms from an average of 7 pigs/farm to 15 pigs/farm. The last Sardinian Eradication Plan of 2015–2018 (ASF-EP15/18), adopted in December 2012, but fully implemented by 2016, confirmed the banning of free-range pig keeping (Regional Decree n.69, 18th December, 2012, approved by Decision 2011/807/UE) and imposed biosecurity regulations on outdoor Sardinian farms. Incentives were provided to farmers to ensure respect of biosecurity rules and to abandon illegal practices, while disease awareness-raising campaigns were also carried out. Previous studies have shown the efficacy of the measures adopted in Sardinia in the last years to contain and eradicate ASF (40). Since 2014 the number of outdoor farms drastically decreased given the measures adopted by the last ASF eradication program (ASF-EP15/18) [https://www.vetinfo.it/j6_statistiche/#/report-pbi/31]. To evaluate a whole epidemic season, this study covers a 10-year period of analysis (2010–2020), as shown in Figure 2. Based on official data recorded in the Italian National Information System for the Notification of Infectious Animal Disease (SIMAN) database, an ASF outbreak in a domestic pig farm is defined as a diagnosed disease event, in accordance with the World Organization for Animal Health (OIE) Manual of Diagnostic Tests (27). As described in our past studies (22, 40, 42), after some years of few outbreaks occurrence (2006–2009), since 2010 the number of outbreaks in both wild boar and domestic pig populations increased, peaking in 2013 and then decreasing in 2015 (Figure 2). In September 2018, the virus was detected for the last time in domestic pigs, while the last PCR-positive illegal free-ranging pigs and wild boar were detected in January 2019 and April 2019, respectively (26, 37, 39). Since then, 42 seropositive cases in wild boar have been reported as outbreaks.

### Free-Ranging, Outdoor, and Indoor Sardinian Farms

Considering the lack of a specific legislative and universal definition for outdoor farms (31) and given that the outdoor farm definition provided by the working document SANTE/7113/2015 does not fully fit outdoor pig farms in Sardinia, in this work, we provided a definition for each of the three farm type characterizing the Sardinian pig-breeding systems. In this paper, we refer to:

(i) Illegal free-ranging pigs: animals kept permanently outdoors, not fenced, with unlimited access to fields, pastures, forest or woodlands, without buildings or shelters, without an official and clearly defined ownership, neither registration in the BDN. As above stated, these farms type are illegal in Sardinia;
(ii) Outdoor farms: commercial or non-commercial farms where domestic pigs are bred in the open-air, with access to fields pasture limited by concrete-fences with buildings or shelters for feeding or rest, with a defined ownership registered in the BDN;
(iii) Indoor farms: farms where domestic pigs are bred in closed buildings or shelters, without access to fields pasture, with a defined ownership registered in the BDN.

### African Swine Fever Surveillance in Domestic Pig Farms and Epidemiological Investigation Tool

The surveillance program implemented by the ASF-EP15/18 includes different measures to control the disease, such as outbreaks identification by screening checks or for suspicion of disease (42, 43). With each subsequent ASF outbreak diagnosed, during the stamping out, the veterinary authority (VA) conducts an inspection aimed at identifying the origin of ASFV introduction (Legislative Decree n.54, 20 February 2004). During that inspection, the VA draws up an epidemiological investigation and uploads it to SIMAN. Furthermore, according to ASF-EP15/18 rules, all farms located in a radius of 3 km and 10 km around outbreak location are included in “protective” and “surveillance” zones, respectively. The validity period of these zones is defined by regional decrees, which establish a start-end period during which animal movements are not allowed except under specific permission for slaughtering (Legislative Decree n. 54/2004 available at http://www.camera.it/parlam/leggi/deleghe/04054dl.htm). For the present study, all the available epidemiological investigations were collected from SIMAN and evaluated based on inclusion/exclusion criteria. An essential inclusion criterion was the presence of a farm code referring to the epidemiological investigation, with a corresponding entry in the BDN, in order to exclude all outbreaks occurring in wild porcine without a clear ownership (i.e., wild boar, pigs found dead, illegal outdoor pigs). The epidemiological investigation report includes specific session about farm data (i.e., location and owner), animals bred (i.e., type of production, number of animals by species), animal census by categories, farm network (i.e., number and type of the relationships with other farms), animal movements, external visits in farm (i.e., veterinarians, breeders, salesmen), clinical evaluation (i.e., number of dead and symptomatic pigs, date of disease suspicion, date of first symptom, type of symptoms, number of pigs serologically and virologically tested, number of pigs detected as ASFV positive and ASFV antibody positive). Specific session about epidemiological context included the presence/absence of wild boar near to the farm, the most probably origin of the contagion. If the veterinarian suspected that the virus introduction was associated to previous outbreak (i.e., supposing epidemiological correlation) the farm codes of the origin outbreak were reported. The outbreaks were defined as primary or secondary cases based on the European Commission Decision 2003/422/CE. An *ad hoc* database in an electronic closed-response data collection instrument (Microsoft Access, Microsoft Corporation, Redmond, WA, USA) was created. The list of variables collected is reported in Supplementary Table 1.

### Statistical Analysis

Data quality and completeness were tested, and an extensive data check was carried out to evaluate the correspondence between census data (BDN and SIMAN) and those reported in the epidemiological investigations. To evaluate possible differences in farm characteristics and management between indoor and outdoor farms that could be associated with ASF outbreak development, baseline descriptive statistics were grouped by farm type. Quantitative variables were summarized as mean and standard deviation (SD), or median and interquartile ranges (IQRs) as appropriate, whereas qualitative variables were summarized as frequencies and percentages. To compare qualitative variables, either the Chi-square test or Fisher's exact test were applied. Differences between quantitative variables were assessed by the Kruskal–Wallis non-parametric test. To account for the large number of comparisons and to reduce the likelihood of identifying a statistically significant association by chance, a *p* < 0.01 was considered statistically significant, with *p* values between 0.01 and 0.05 considered indicative of a statistical association but epidemiologically weak. Furthermore, in order to fully evaluate the epidemiological neighbor context of each outbreak occurred, features regarding wild boar, illegal free-ranging pigs and domestic pig farms density have been collected based on a 10 km radius around each outbreak farm. This size has been chosen considering both the maximum radius of ASF surveillance system in domestic pigs (i.e., surveillance zone) and the estimated moving radius of wild boar in Sardinia (44). All the statistical analyses were performed using the open-source R software v.4.0.5 and a *p* < 0.05 was considered as statistically significance.

### Estimation of Possible Transmission Network

Given the need to estimate the ASFV transmission distance more appropriate for the Sardinian rural context two context-specific considerations have been taken into account: (a) the epidemiological investigations collected reported a mean values of distance between primary and secondary cases of 3 km and a maximum value of 10 km, while long-distance transmission (>10 km) are limited; (b) long-distance transmission routes are associated to infected food waste, while most of the illegal animals movements [mainly identified as movement of male for reproduction (42)] occurred in proximity of the farm.

Two probability algorithms based on transmission kernel functions *f* following nearest-neighbor and uniform-kernel-smoothed distribution were applied (45–50). The first would reflect the disease transmission by legal trade or wild boar movements, and thus the unit closest to the secondary case was selected as primary case. For the nearest-neighbor algorithm the transmission distance was limited to a radius *r* estimated based on a Pert distribution ranging from 0.5 to 10 km, and the most probable distance value of 3 km. These values allowed to reflect the legal animal movements based on surveillance and protective zones, and the wild boar movements. Considering that the uniform-kernel attributes equal probabilities, the second transmission kernel takes into account long-distance transmission human mediated. The two algorithms implemented depending solely on the distance between paired outbreaks, under the following assumptions:

(i) The ASF incubation period followed a Pert distribution ranging from 3 to 20 days, with 6 days as the most probable value (51–53).
(ii) The outbreak start date (defined by the ASF suspicion date) associated with a secondary case must be at least equal to the start date of the paired primary case, plus the estimated incubation period for ASFV.
(iii) The onset of the secondary outbreak must begin before the closure/extinction of the primary outbreak, minus the incubation period.
(iv) The outbreak end date was obtained by fitting a Pert distribution and considering 6, 60, and 30 days as the minimum, maximum, and most probable values, respectively (45).

The distributions of the three variables related to the epidemiological neighbor context between indoor and outdoor farms were evaluated. If one or more variables were differently distributed the kernel functions were corrected using these variables as covariates. A smooth searching neighborhood was applied in order to limit the range of the kernel at the 95% confidence interval (95% CI). A weight *wij* of each primary *i* and secondary *j* case pair was assigned to the respective value of the kernel function *f* (*i,j*|°), if the assumptions above were not violated. Otherwise, the weight *wij* was set to zero. Normalizing correction was applied to define the transmission probabilities *pij* for each primary case as:

$$p_{ij}=\frac{w_{ij}}{\sum_{i=1}^{N}w_{ij}},\;j\;=\;1\ldots N$$

The secondary case is linked to a single primary case by sampling from a binomial distribution. When *pij* = 0, linkage between primary and secondary case does not occur (i.e., the primary outbreak). Binomial sampling was used to build the transmission network. Once a primary case was identified, no other primary cases could be linked to its associated secondary cases (54). When a network transmission cluster was identified, the ASFV transmission distance was calculated from each secondary case using latitude and longitude coordinates to the centroid of the cluster, and reported in kilometers.

Finally, we calculated the time difference (*delta_time*) between each secondary case and its associated primary case, and described this time as mean (95% CI). The transmission networks resulting from both the applied kernel functions were compared to the epidemiological correlation network reported in the official epidemiological reports. The agreement between the two network described by the algorithms and the network described by the epidemiological investigations in identify the secondary cases was first evaluated in a contingency table. The degree of accuracy and reliability in secondary cases classification was evaluated applying the Cohen's kappa coefficient (55), and the 95% CI were calculated by the method proposed by Fleiss et al. (55). The kappa coefficients were evaluated using the guideline outlined by Landis and Koch (56), where the strength of the kappa coefficients is slight if *K* = 0.01–0.20; fair if *K* = 0.21–0.40; moderate if *K* = 0.41–0.60; substantial if *K* = 0.61–0.80 and almost perfect if *K* = 0.81–1.00. This comparison would provide an evaluation of the ability of the VA in detect illegal trade and well-traceability of the contact between farms. Even if the secondary case definition does not perfectly match the basic reproduction number, which is the average number of secondary cases due to the introduction of a primary case in a completely susceptible population (57–61), the secondary case estimation could be interpreted as a proxy able to quantify the spread of an infection predicting its speed. Thus, the epidemic is in decline if the average of secondary cases is ≤1 and on the rise if >1 (61). In order to test the hypothesis of ASFV transmission decline following the control measures against illegal free-ranging pigs fully implemented in 2016 (39), comparisons in the number of secondary cases before/after this period are presented. Finally, characterizing the number of “faster than average” spreading outbreaks matter, vs. “normal outbreaks” is of great concern in order to evaluate if these outbreaks were also associated with larger number of secondary cases, increasing the geographical spread and making harder the application of efficacy control measures. Preliminary data evaluation regarding the number of secondary cases and the number of “*fast*” outbreaks generated by each cluster was performed by graphical tool and Pearson correlation coefficient. Kernel functions and transmission networks were implemented in open-source R software v.4.0.5 and Q-Gis v.3.18.3.

### Multivariable Analysis

To evaluate which farm characteristics could have contributed to speed up the ASFV transmission, each secondary outbreak was classified as “*normal*” or “*fast*” based on the delta_time value. Considering the reported outbreak as the epidemiological unit, the outbreak was defined as “*normal*” if the delta_time was equal or higher the mean value, “*fast*” if lower. Two logistic mixed models (62) were fitted based on the two kernel transmission networks. The final aim of these models was to establish the main farm' characteristics involved in the probability of observing an untimely outbreak respect to on-time outbreak. Correlation coefficients between variables were calculated using Spearman non-parametric test, multi-collinearity between variables was tested (63) and the variance inflation factor (VIF) > 2 was used to identify and delete potentially redundant features (64, 65). Assuming that the observations between years and clusters were not independent, we applied a logistic multilevel mixed model, including the year and the cluster as random effects to control the between-year and cluster differences. Given that no outbreak reoccurrence was identified in the same farm, no random effect associated with the farm was included. Risk factors selection was performed by a stepwise selection process (66), and the best fitting was established based on adjusted R2, and Akaike's information criterion (AIC) (67) values. Considering the completely absence of quarantine of new animals in outdoor farms, the role of this variable was evaluated as confounding factor. Thus, the inclusion in the final multivariable models of quarantine and/or type of farm as explicative variables was evaluated by AIC value. The logistic multilevel mixed model results were presented as adjusted odds ratio (OR_adj_) calculated with the logistic regression method (61). Often model validation is performed using data referred to some years as training dataset, and the rest as test dataset. Considering the need of including the “year” as random effect, the model validation was performed on random selected groups of data, with 1:1 proportion.

## Results

From 2010 to 2020, a total of 1068 ASF outbreaks were reported in Sardinia in all the target populations (i.e., wild boar, domestic and outdoor pigs) (Figure 2). Of these, 695 outbreaks were excluded from our analysis because ASFV genome or ASF antibodies were detected in wild boar and 68 because outbreaks occurred in illegal free-ranging pigs. Thus, considering the main aim of this study, only the 305 outbreaks occurred in domestic pig farms from 2010 to 2018 were included. Farms were indoor or outdoor, each associated with a farm code recorded in the BDN (Table 1). Of these 305 outbreaks, 48% (147) occurred in outdoor farms, while 52% (158) in indoor farms (Figure 1C). Most of the outbreaks occurred in 2012 (69, 23%) and 2013 (34%), specifically in May (72, 24%) and June (61, 20%). Considering that the “reproduction period” is the phase in which piglets destined for fattening and replacement are produced, and the “fattening period” is the production of pigs for slaughter, farms were identified as (1) “*close-cycle breeding*”, referring to those farms where both phases are carried out, (2) “*open-cycle breeding*” where only one phase is carried out (i.e., reproduction or fattening), (3) “*for self-consumption*”, not intended for selling but for self-consumption by the farm holder and his household (Table 1). The infected farms were mainly closed-cycle production in both indoor (128, 81%) and outdoor (144, 98%) farms, with the median number of bred pigs being 19 (IQR = 11–28 and 5–23, respectively). Sows were the main animal category, with a median value of 3 (IQR = 2–6) and 4 (3–9) in indoor and outdoor farms, respectively (Table 1). Similar distributions were found between indoor and outdoor farms for management characteristics such as distance from other farms, slaughterhouse within the farm, loading and unloading facilities, animal identification and compilation of the farm's register, disinfection, disposal clothing and feeding pigs with kitchen waste.

**Table 1:** Baseline descriptive statistics of the farm features, by farm type (indoor and outdoor).

**Variables**	**Outbreaks in indoor farms (*n* = 158)**	**Outbreaks in outdoor farms (*n* = 147)**
**Data of ASF outbreak**		
2010 2011 2012 2013 2014 2015 2016 2017 2018	5 (3%) 16 (11%) 29 (18%) 31 (19%) 32 (20%) 11 (7%) 14 (9%) 15 (10%) 5 (3%)	2 (1%) 15 (10%) 40 (27%) 72 (49%) 8 (6%) 5 (3%) 4 (3%) 1 (1%) 0 (0%)
**Production type**		
Closed-cycle Open cycle Self-consumption	128 (81%) 5 (3%) 25 (16%)	144 (98%) 3 (2%) 0 (0%)
**N. of animals**	15 [11–28]	15 [5–23]
**N. of animals by categories**		
Boar Sows Hogs Piglets	1 [1–1] 3 [2–6] 1 [0–4] 0 [0–10]	1 [1–2] 4 [3–9] 1 [0–3] 1 [1–9]
**Distance from other farms (meters)**		
<500 m 500–1,000 m >1,000 m	107 (68%) 37 (23%) 14 (9%)	109 (72%) 35 (26%) 3 (2%)
Declared relationship with other farms***		
Family relationship Working collaboration No relationship	71 (45%) 25 (16%) 62 (39%)	88 (60%) 47 (32%) 12 (8%)
**Presence of slaughterhouse in farm**		
Yes Not	2 (1%) 156 (99%)	0 (0%) 147 (100%)
Type of fence***		
Double fence Single solid fence Single metal fence Not fenced	33 (21%) 78 (49%) 30 (19%) 17 (11%)	15 (10%) 22 (15%) 93 (63%) 17 (12%)
Suspected contact with wild boar**		
Yes Not Not specified	44 (28%) 101 (64%) 13 (8%)	66 (45%) 74 (50%) 7 (5%)
Shelter***		
Open Close	30 (19%) 128 (81%)	104 (71%) 43 (29%)
**Loading and unloading**		
Inside farm Outside farm	73 (46%) 85 (54%)	66 (45%) 81 (55%)
Quarantine new animals***		
Yes Not	142 (90%) 16 (10%)	0 (0%) 147 (100%)
**Animal identification**		
Yes Not	123 (78%) 35 (22%)	135 (92%) 12 (8%)
**Farm register compiled**		
Yes Not	115 (73%) 43 (27%)	110 (75%) 37 (25%)
**Disinfection**		
Yes Not	126 (80%) 32 (20%)	100 (68%) 47 (32%)
**Disposable clothing**		
Yes Not	19 (12%) 139 (88%)	0 (0%) 147 (100%)
Animal separation by categories***		
Yes Not Not specified	57 (36%) 68 (43%) 33 (21%)	7 (5%) 37 (25%) 103 (70%)
Storage of livestock waste/manure/uneaten food***		
Yes Not	122 (77%) 36 (23%)	50 (34%) 97 (66%)
Carcass storage***		
Incineration in farm Burial in farm Not stored/specified	14 (9%) 118 (75%) 26 (16%)	4 (3%) 36 (24%) 107 (73%)
**Biting pigs with kitchen waste**		
Yes Not Not specified	33 (21%) 119 (75%) 6 (4%)	25 (17%) 115 (78%) 7 (5%)
Farmer as a hunter**		
Yes Not	36 (23%) 122 (77%)	60 (41%) 87 (59%)
**ASF tested animals**		
PCR+ /Ab+ PCR+/Ab– PCR–/Ab+ PCR–/Ab–	2 [1–5] 1 [1–4] 1 [0–1] 5 [2–15]	1 [1–4] 1 [1–3] 1 [0–2] 6 [4–10]
**Virus isolation**	38 (24%)	34 (23%)
**Days for outbreak confirmation** (from suspicion data)	5 [2–11]	8 [4–12]
**Days for stamping out** (from suspicion data)	7 [4–9]	10 [5–13]
**N. died animals**	1 [1–3]	1 [1–3]
**N. animals with symptoms**	2 [1–4]	1 [1–3]
**Epidemiologically correlated**		
Yes Not Not specified	78 (49%) 64 (40%) 16 (11%)	85 (58%) 47 (32%) 15 (10%)
**Hypothesized origin of contagious**		
Contact with wild boar Human factor Unknown	28 (18%) 113 (71%) 17 (11%)	73 (50%) 59 (40%) 15 (10%)

Data are presented as mean (standard deviation), median [I-III quartile], frequency (percentage). Statistically significant differences are identified by a p-value < 0.0001 (***), or p-value between 0.05–0.0001 (**).

Considering the overall population of Sardinian farms during each year in study (mean = 16,671, SD = 756), of which about the 85% (mean = 14,456, SD = 927) were indoor and the 15% (mean = 2216, *SD* = 415) wereoutdoor farms, the baseline probability to be infected was six times more in outdoor farms than indoor farms (OR = 6.069, 95% CI = 4.827–7.631, *p* < 0.0001). Statistically significant differences between outdoor and indoor farms were detected in the declared relationship with other farms, reported as working collaboration or familiar relationship (i.e., father, son, cousins, etc.) [$\chi_{\left(1,\;\text{N}\;=\;305\right)}^{2}$ = 41.98, *p* < 0.0001], type of fence [$\chi_{\left(1,\;\text{N}\;=\;305\right)}^{2}$ = 70.07, *p* < 0.0001], suspected contact with wild boar [$\chi_{\left(1,\;\text{N}\;=\;305\right)}^{2}$ = 9.98, *p* = 0.007], type of shelter [$\chi_{\left(1,\;\text{N}\;=\;305\right)}^{2}$ = 82.83, *p* < 0.0001], application of quarantine for the new animals [Fisher (1, *N* = 305) = 0.0001, *p* < 0.0001] and their separation by categories [$\chi_{\left(2,\;\text{N}\;=\;305\right)}^{2}$ = 83.95, *p* < 0.0001], storage of livestock waste/manure/uneaten food [$\chi_{\left(1,\;\text{N}\;=\;305\right)}^{2}$ = 57.79, *p* < 0.0001], the concomitant role of the farmer as a hunter [$\chi_{\left(1,\;\text{N}\;=\;305\right)}^{2}$ = 11.48, *p* = 0.0007] and carcass storage [$\chi_{\left(1,\;\text{N}\;=\;305\right)}^{2}$ = 98.27, *p* < 0.0001]. Laboratory tests revealed that, during the outbreaks, the median number of pigs in the initial phase of the disease (ASFV positive and ASF antibody negative) was 2 (IQR = 1–5) in indoor farms and 1 (IQR = 1–4) in outdoor farms. A median value of 1 pig (IQR = 1–4 and 1–3, respectively) that developed antibodies during virus replication (ASFV positive and ASF antibody positive) was recorded in both types of farms. A median of 1 animal (IQR = 0–1 and 0–2, respectively) survived the disease and tested ASFV negative and ASF antibody positive. The virus was isolated by Malmquist or immunofluorescence laboratory tests in about 24% of the outbreaks, with a median value of 1 dead pig (IQR = 1–3). A median of 2 pigs (IQR = 1–4) and 1 pig (IQR = 1–3) showed common symptoms in indoor and outdoor farms, respectively. Overall, 70% (213) of the outbreaks were recorded after symptoms were reported, mainly by the farmer (78%, 166) or veterinarians (22%, 47). Common symptoms were anorexia, hemorrhage, fever, loss of appetite, non-coordinated movements, dyspnoea, cyanosis, fatigue, abortion, diarrhea, epistasis, haematuria, and cough. In 59% (180) of the outbreaks, the farmer reported disease suspicion after moderate symptoms; in 17% (52) of the outbreaks, the farmer reported disease suspicion after sudden death in combination with other symptoms; and 24% (73) of the outbreaks were either not reported by the farmer (i.e., disease reporting by veterinarians) or were reported by the farmer to the veterinarian only after the death of a second pig. A median of 5 (IQR = 2–11) and 8 (IQR = 4–12) days from the ASF suspicion date was necessary to confirm the disease suspicion based on the OIE Diagnostic Manual for indoor and outdoor farms, respectively. Furthermore, even more days [7 (IQR = 4–9) and 10 (IQR = 5–13)] from the ASF suspicion date were necessary to apply stamping-out measures. According to the epidemiological investigation carried out by the veterinarians, the virus introduction in indoor farms was mainly associated (113, 71%) with human activities (i.e., people's movements between farms, uncontrolled animal introduction, low biosecurity, inadequate disinfection, or kitchen waste), followed by the contact with wild boar for pigs belonging to the outdoor farms. Otherwise, the contact with wild boar seems to be the first way of ASFV introduction in outdoor farms (73, 50%).

**Figure 3:**
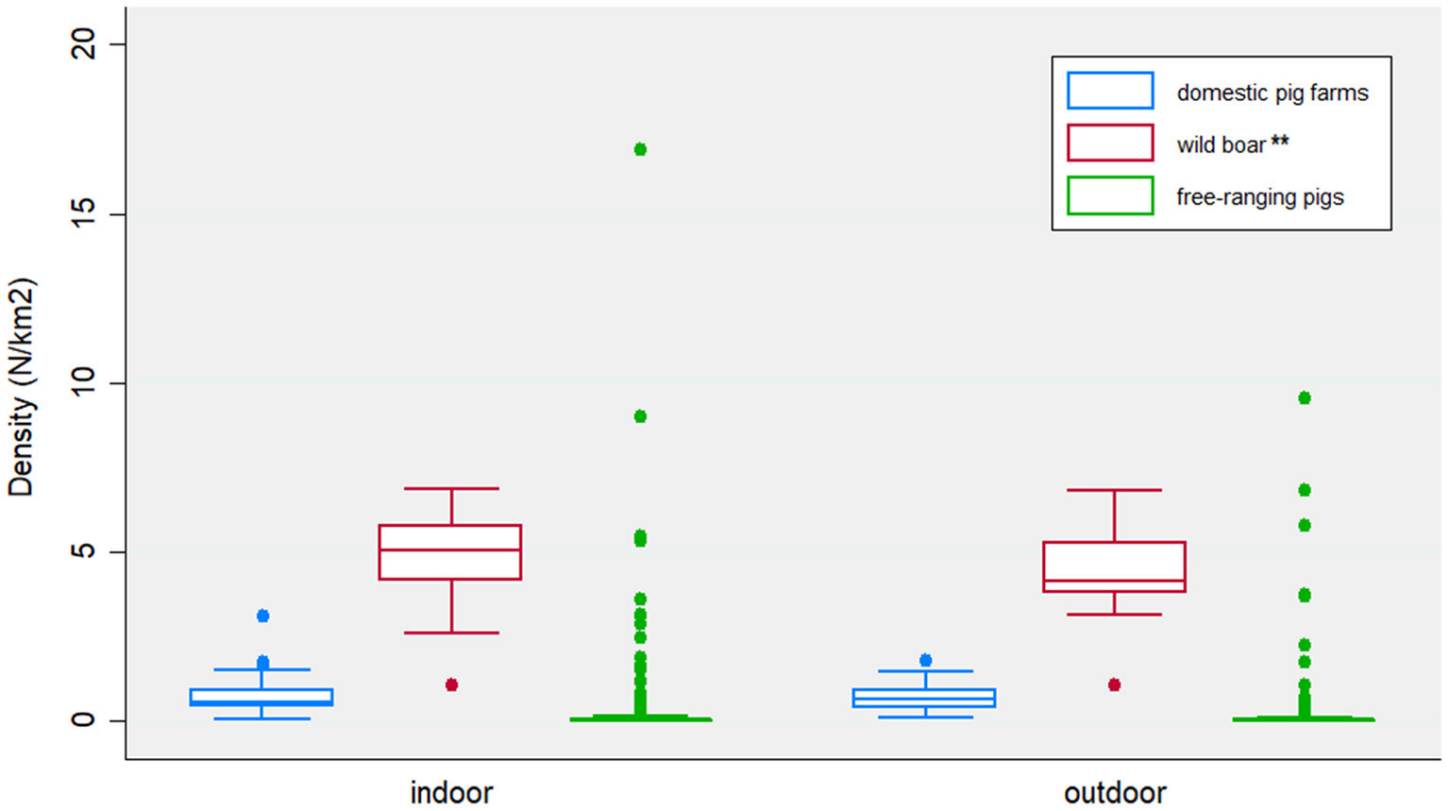
Distribution of the density of domestic pig farms, wild boar and free-ranging pigs, expressed as number/km^2^, by indoor and outdoor farms. Features were collected based on 10 km^2^ radius around each epidemiological unit (i.e., outbreak farm). Statistically significant differences between indoor and outdoor farms are identified by a *p*-value <0.0001 (***), or *p*-value between 0.05–0.0001 (**).

The VA defined as epidemiologically correlated a total of 78 (49%) outbreaks occurred in indoor farms and 85 (58%) in outdoor farms. The features related to the epidemiological context are reported in Figure 3. The density of wild boar is statistically significant higher (*p* = 0.0004) around indoor (mean = 4.95/km^2^, *SD* = 1.03) rather than outdoor farms (mean = 4.56/km^2^, *SD* = 0.89). Otherwise, similar distributions of domestic pig farms and illegal free-ranging pigs have been detected in indoor and outdoor farms.

### Nearest-Neighbor Kernel Transmission Network

**Figure 4:**
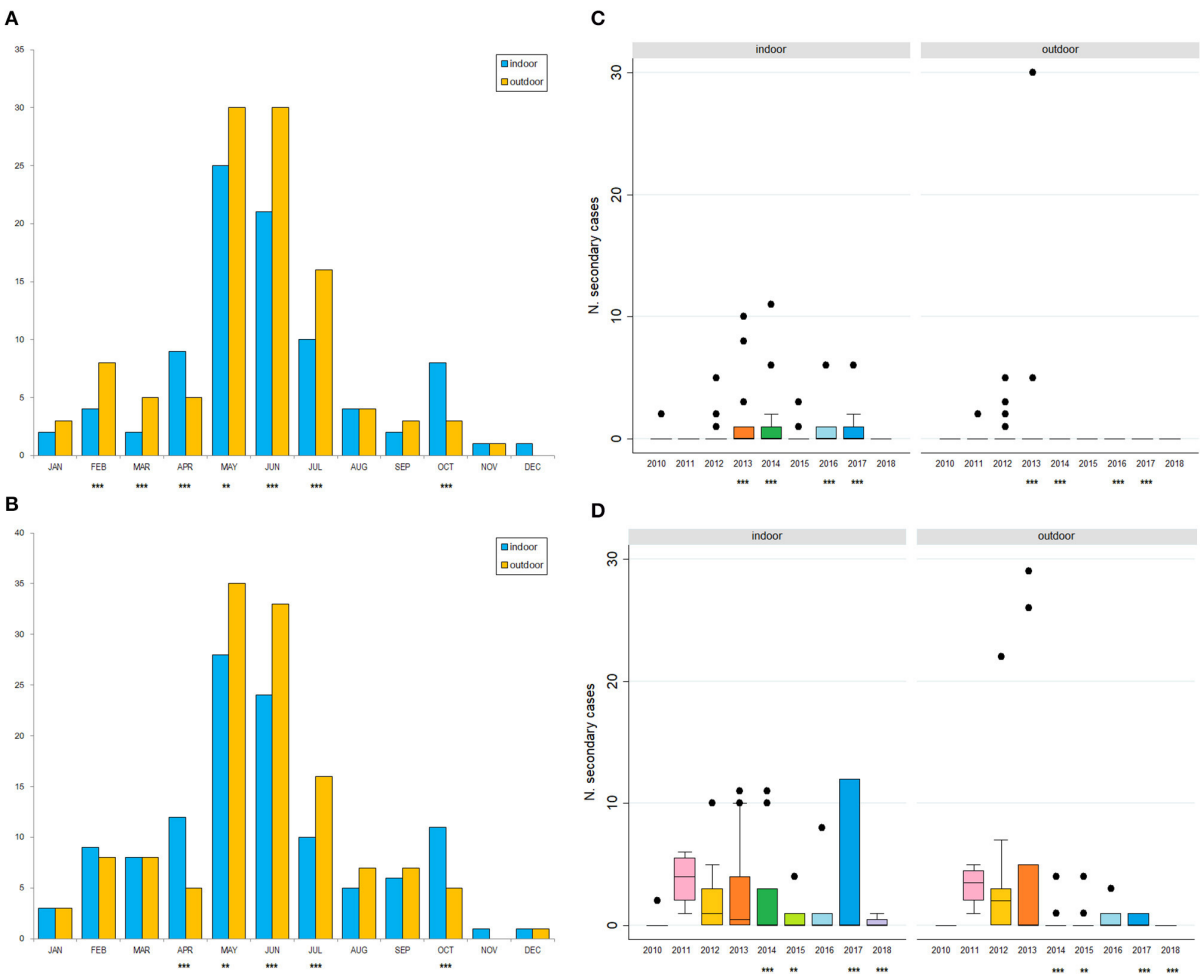
Number of the secondary cases by year distribution **(A–C)** and overall month distribution **(B–D)** from 2010 to 2018. Data are represented by the number of secondary cases defined by nearest-neighbor **(A,B)** or uniform-smoothed kernel functions **(C,D)**. Statistically significant differences between indoor and outdoor farms are identified by a *p*-value <0.0001 (***), or *p*-value between 0.05–0.0001 (**).

**Figure 5:**
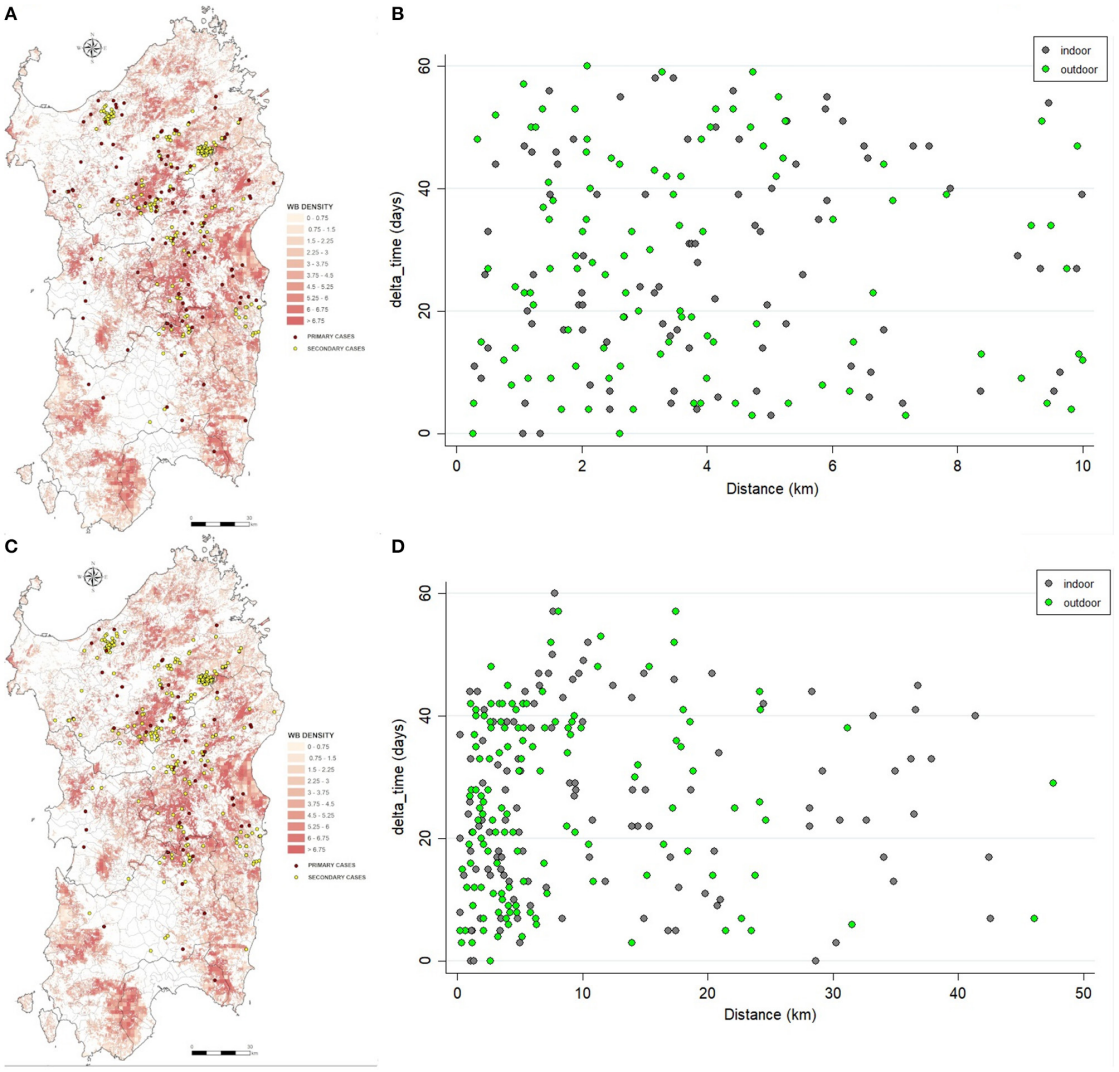
Spatial distribution of the primary (red) and secondary cases (yellow) detected by nearest-neighbor **(A)** or uniform-smoothed kernel functions **(C)**. Scatterplots shown the relation between difference time and distance between the secondary outbreak and its primary outbreak, by nearest-neighbor **(B)** or uniform-smoothed kernel functions **(D)**.

**Figure 6:**
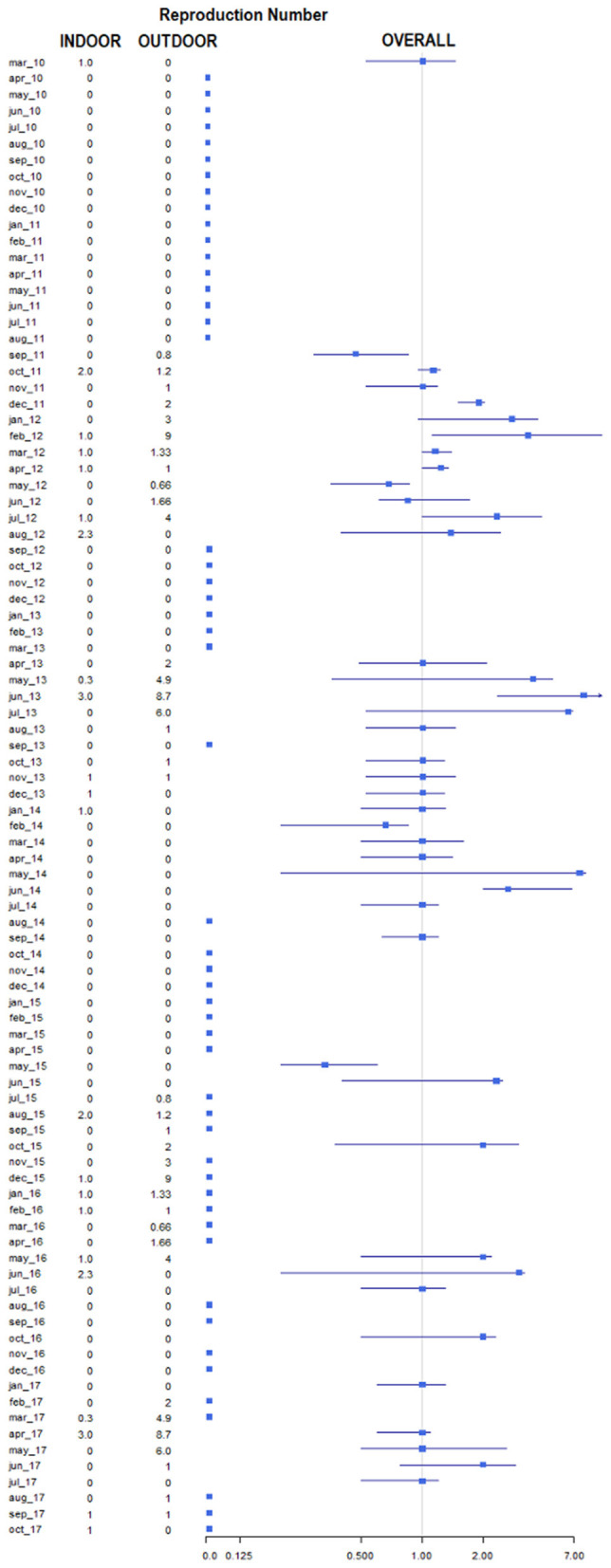
Forestplot representing the average number of secondary cases (reproduction number) by month and year. Data are reported as overall (squares represent the estimates, lines indicate the 95% confidence intervals), average number in indoor and outdoor.

**Figure 7:**
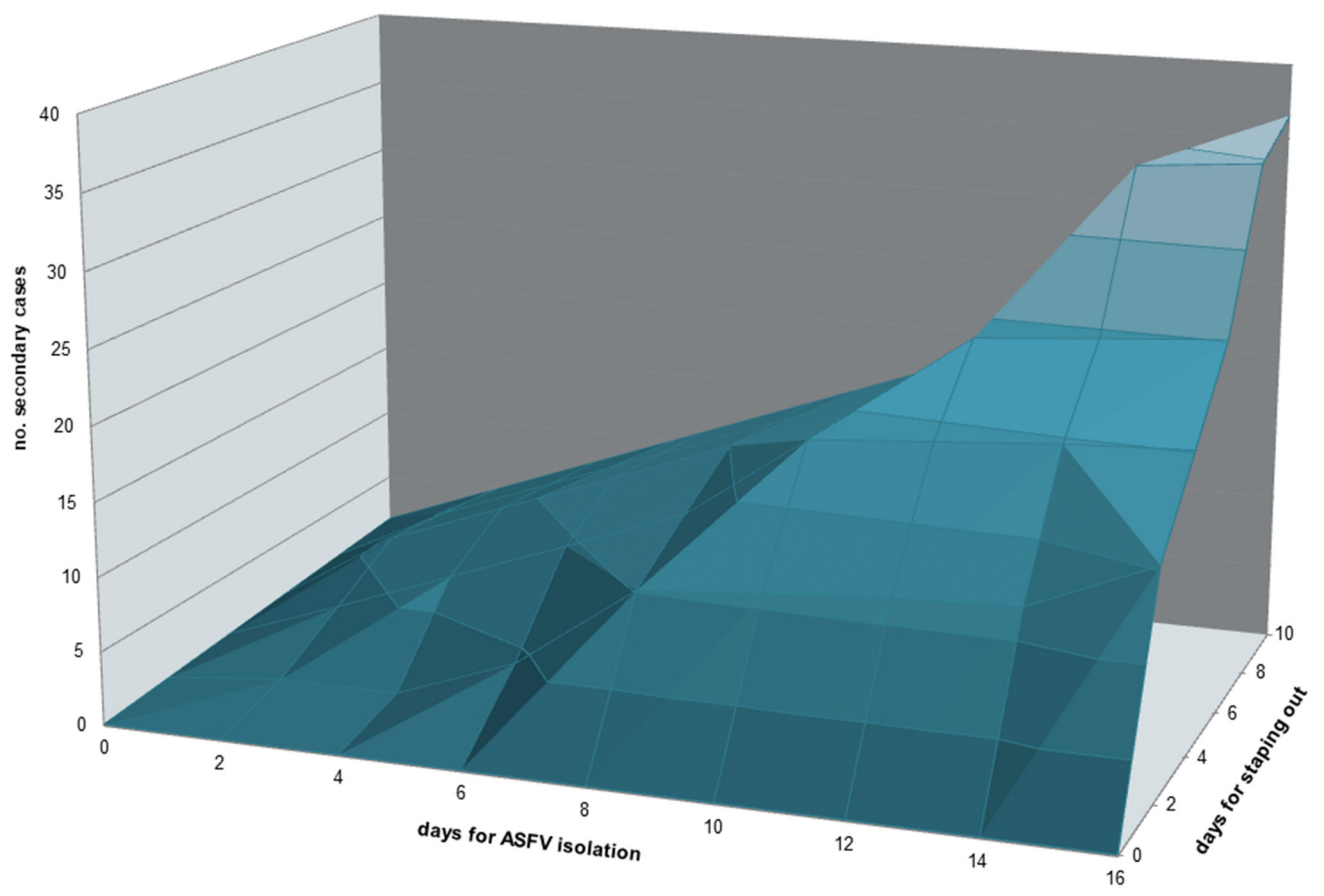
Contour plot representing the relation between time (days) used for ASFV isolation and time (days) used for stamping out in the primary outbreak with the number of secondary cases associated.

Of the 305 outbreaks, 108 primary cases occurred from 1st January 2010 to 10th September 2018. In addition, 197 secondary cases were generated, mainly in 2012 (44, 22%), 2013 (88; 45%). The average number of secondary cases was 0.5 (95% CI = 0.0–0.9) in 2010 and increased to 1.9 (95% CI = 0.1–2.6) in 2013 (Figure 4A). In particular, most of the secondary cases occurred during May (55, 28%) and June (51, 26%) each year (Figure 4B). Figure 5A reports the nearest-neighbor transmission network of ASF spread among infected domestic pig farms in Sardinia. The estimated mean transmission distance was 3.87 km (95% CI = 3.51–4.23), and the average time interval (delta_time) was 16 days (95% CI = 14.3–20.6) between paired cases (Figure 5B). Overall, from each primary case, a mean of 1.86 (95% CI = 1.62–2.82) secondary cases was generated. Disease transmission drastically reduced from the second half of 2017 (average number of secondary cases <1) (Figure 6). No outbreaks, neither primary nor secondary, occurred in registered pig farms after September 2018. Worth to highlight that the number of secondary cases increased when the time needed for both virus isolation and stamping out increased as shown in Figure 7: after 4 days for virus isolation and 5 days for stamping out, each day of delay corresponded to a doubling of secondary cases. The number of secondary cases associated with primary indoor farms or primary outdoor farms was similar with a mean of 0.45 (95% CI = 0.32–0.58) and 0.68 (95% CI = 0.51–0.84), respectively. Three main clusters arose from outdoor farms located in Bitti (2012), Padru (2013) and Bulzi (2013). The primary case of these clusters generated 17, 39 and 36 secondary cases, infecting about 10% of the total farms located in the radius, and the spread of the disease spanned 44, 58, and 55 days, respectively. The epidemiological landscape of these three clusters was similar, with an average farm density of 10 farms/10 km^2^ (*SD* = 7 farms/km^2^) and an outdoor farm density of about 5 farms/10 km^2^ (*SD* = 4 farms/km^2^), in which a median of 11 (IQR = 6–30) pigs were bred. In the first cluster of Bitti, most of the secondary cases (15, 88%) occurred in outdoor farms, with an average of 5 (*SD* = 1.2) symptomatic pigs reported in each outbreak. Similar percentage of secondary cases in outdoor farms was reported in Padru and Bulzi, but with significantly lower average of symptomatic pigs (mean = 1.6, *SD* = 0.5; mean = 0.5, *SD* = 0.03, respectively). Furthermore, in the area where the Bitti and Padru clusters occurred the presence of illegal free-ranging pigs had historically been reported, while these animals were never detected in the hunting management unit of Anglona-Gallura, where Bulzi is located (22).

### Uniform-Kernel-Smoothed Transmission Network

A total of 60 primary and 245 secondary cases were detected by uniform-kernel-smoothed transmission network. Most of the secondary cases occurred in 2012 (60, 24%) and 2013 (93, 38%), particularly in May (63, 26%) and June (57, 23%). The average number of secondary cases was 0.2 (95% CI = 0.0–0.59) in 2010 and increased to 4.35 (95% CI = 0.59–8.11) in 2013 (Figures 4C,D). Figure 5C reports the nearest-neighbor transmission network of ASF spread among infected domestic pig farms in Sardinia. The estimated mean transmission distance was 11.2 km (95% CI = 9.91–12.35), and the average time interval (delta_time) was 20 days (95% CI = 17.4–22.5) between paired cases (Figure 5D). Overall, from each primary case, a mean of 4.16 (95% CI = 3.09–5.23) secondary cases was generated. The number of secondary cases associated with primary indoor farms or primary outdoor farms was similar with a mean of 2.01 (95% CI = 1.14–2.88) and 2.11 (95% CI = 0.64–3.58), respectively. The estimated average number of secondary cases over the first 6 years in this study is significantly higher with respect to that estimated in 2016–2018, (μ_2010−2015_ = 0.98, SD_2010−2015_ = 0.35, μ_2016−2018_ = 0.43, SD_2010−2015_ = 0.24, *p* < 0.001), indicating a reduction in ASF spread and disease extinction in domestic pigs, given that the number of secondary cases is equal or lower than 1 since 2017. As well as in the nearest-neighbor transmission network, the same three main clusters arose from outdoor farms located in Bitti (2012), Padru (2013), and Bulzi (2013) were detected. Both the fitted kernels were adjusted for the wild boar density given its different distribution between the two types of farms (indoor and outdoor), as reported in Table 2. An exponentially increasing intensity of secondary cases with increasing wild boar population density values when the population density is expressed as a log is represented in Supplementary Figure 1.

**Table 2:** Descriptive statistics of the epidemiological context features based on 10 km of radius around each outbreak farm, by farm type (indoor and outdoor).

**Variables**	**Outbreaks in indoor farms**	**Outbreaks in outdoor farms**
	**(*n* = 158)**	**(*n* = 147)**
Wild boar density (km^2^)**	4.95 (1.03)	4.56 (0.89)
Domestic pig farms density (km^2^)	0.71 (0.42)	0.72 (0.35)
Illegal free-ranging pigs density (km^2^)	0 [0–0.8]	0.3 [0–0.4]

Data are presented as mean (standard deviation), median [I-III quartile]. Statistically significant differences are identified by a p-value < 0.0001 (***), or p-value between 0.05–0.0001 (**).

### Transmission Networks' Agreement

The degree of accuracy and reliability of epidemiological investigation tools was estimated based on the two kernel transmission networks. Comparisons were applied only for 274 outbreaks where the origin of ASFV introduction or specific epidemiological correlation were detailed in the veterinary reports. The ability of the epidemiological investigation reports to correctly detect secondary cases in accordance to the kernel transmission models is reported in Table 3. The epidemiological investigations reported 111 primary cases and 163 secondary cases. In comparison with nearest-neighbor kernel transmission network, 89 primary and 154 and secondary cases were equally identified with a substantial agreement of 89.9% (Cohen's *k*: 0.76, 95% CI =0.68–0.84). In comparison with uniform-kernel-smoothed transmission network, the epidemiological investigation reports agreed in defining 55 and 158 primary and secondary, respectively, with a moderate agreement of 77.8% (Cohen's *k*: 0.50, 95% CI =0.39–0.61). Thus, epidemiological investigations carried out by veterinarians are more able to detect small-distance secondary cases rather than ASFV long-distance transmissions. A focus on the false-secondary cases identified underlines that the five incorrectly secondary cases of the uniform kernel occurred after a period longer than 70 days from the previous outbreaks, and thus excluded as secondary cases for the models assumptions. These outbreaks associated with human-mediated spread occurred in 2011 in indoor farms in Oristano Province (Central-West Sardinia) and in 2017 in Cagliari province. The epidemiological investigation reports specified that these were generated through frozen infected food waste from Central-East Sardinia.

**Table 3:** Agreement table matrix.

		**Epidemiological investigation**			
		**Primary**	**Secondary**	**Total**	**Agreement**
**Nearest-neighbor kernel**	**Primary**	89	9	98	Substantial agreement (89.9%), Cohen's k: 0.76 (95% CI =0.68–0.84)
	**Secondary**	22	154	176	
**Uniform kernel**	**Primary**	55	5	60	Moderate agreement (77.8%), Cohen's k: 0.50 (95% CI =0.39–0.61)
	**Secondary**	56	158	214	
	**Total**	111	163	**274**	

*Agreement values are presented as overall agreement frequency, Cohen's kappa coefficients and 95% confidence intervals (95% CI)*.

### Characterizing the Faster ASF Outbreaks

**Figure 8:**
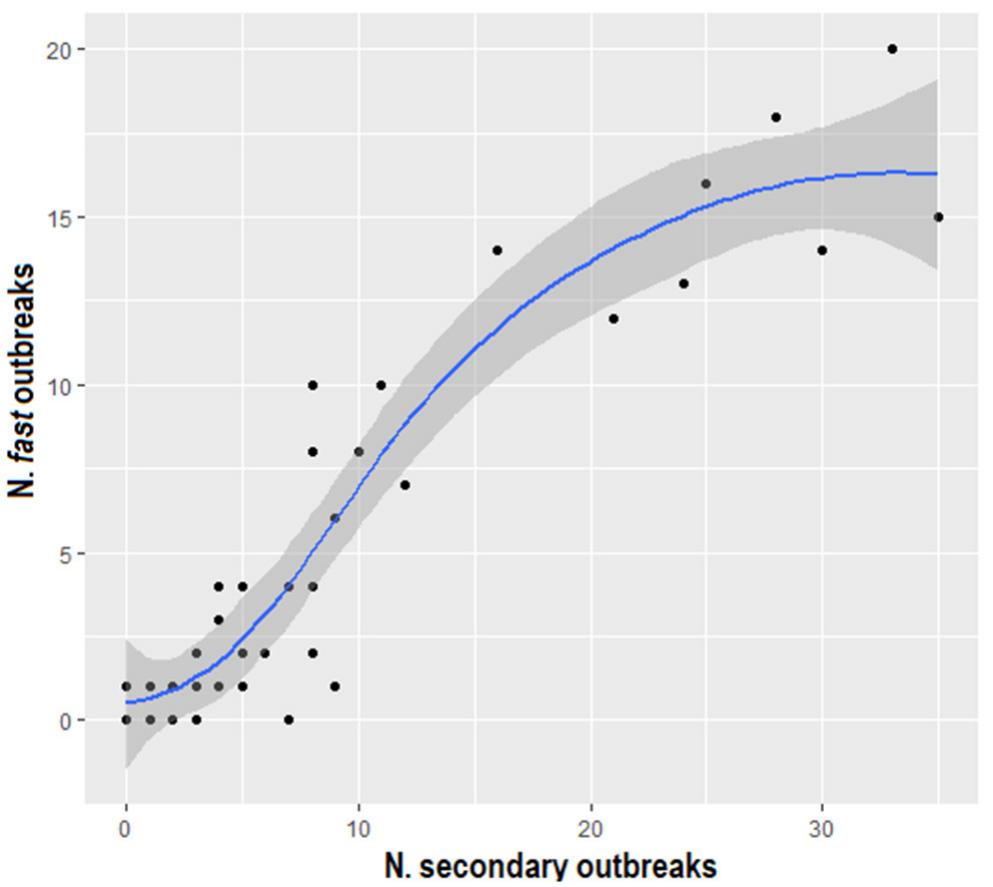
Scatter plot displaying the number of fast outbreaks versus the number of secondary outbreaks, using both the nearest-neighbor and the uniform kernel functions.

The outcome defined by delta_time values generated by nearest-neighbor and uniform-kernel-smoothed function characterized 135 “*normal*” and 62 “*fast*” outbreaks, and 154 “*normal*” and 94 “*fast*” outbreaks, respectively. The graph in Figure 8 suggests a linearly increasing relationship between the number of secondary cases and the number of “*fast*” outbreaks using both the kernel functions. Furthermore, the Pearson's correlation coefficient equal to 0.916 (*p* < 0.0001) confirms this association.

The logistic mixed model results fitted on the delta_time values generated by nearest-neighbor kernel function highlighting the main features associated with the probability of observing a “*fast*” outbreak with respect to a “*normal*” outbreak (Table 4). This probability was 1.36 times more in outdoor farms compared to indoor farms (OR_adj_ = 1.36, 95% CI = 1.12–3.77, *p* = 0.044) and approximately three times more if the farm was not fenced (OR_adj_ = 2.65, 95% CI = 1.90–3.69, *p* < 0.0001). Furthermore, the probability to observe speed outbreak increase of 8.56 times if the farm was located a distance <3.87 (mean value of the network) from the centroid of the cluster (OR_adj_ = 8.56, 95% CI = 4.90–14.98, *p* < 0.0001). Increasing by one the number of days needed for outbreak confirmation and the number of symptomatic pigs, the probability of “*fast*” outbreak occurrence increased of 3% (OR_adj_ = 1.03, 95% CI = 1.01–1.05, *p* = 0.004) and 8% (OR_adj_ = 1.08, 95% CI = 1.02–1.11, *p* = 0.005), respectively. Otherwise, the fast outbreak probability was about half when <10 pigs were breed in the farm (OR_adj_ = 0.53, 95% CI = 0.31–0.89, *p* = 0.016), when no relationship with other farms were detected (OR_adj_ = 0.49, 95% CI = 0.25–0.96, *p* = 0.028), or when no hunting activities by the farmer (OR_adj_ = 0.45, 95% CI = 0.25–0.83, *p* = 0.002) are reported in epidemiological investigation reports. Finally, the probability of observing “*fast*” outbreak decreased of about 80% in farms that declare disposal clothing usage (OR_adj_ = 0.81, 95% CI = 0.70–0.94, *p* = 0.005). No year random effect was included given the lower AIC associated with the model which excluded this effect (AIC = 1,754.33, delta AIC = 17.57).

**Table 4:** Logistic mixed model results using fast (*y*=1) or normal (*y*=0) outbreaks as outcome, with a cut-off <16 or ≥16 days from primary case for categories definition, based on Nearest-neighbor kernel function.

**Outcome = fast outbreak detected by Nearest-neighbor kernel function**			
**Variable**	**OR_**adj**_**	**95% CI**	***p***
**Type of farm**			
Indoor	Ref.		
Outdoor	1.36	1.12–3.77	0.044
**Distance** < 3.87 km	8.56	4.90–14.98	<0.0001
**Days for outbreak confirmation**	1.03	1.01–1.05	0.004
**N. animals with symptoms**	1.08	1.02–1.11	0.005
**Type of fence**	Ref.		
Fenced^§^	Ref.		
Not fenced	2.65	1.90–3.69	<0.0001
**N. pigs**			
>10 ≤10	Ref. 0.53	0.31–0.89	0.016
**Relationship with other farms**			
Yes Not	Ref. 0.49	0.25–0.96	0.028
**Disposable clothing**			
Not Yes	Ref. 0.81	0.70–0.94	0.005
**Farmer as a hunter**			
Yes Not	Ref. 0.45	0.25–0.83	0.002
**Random effect**	**Est**	**SE**	**95% CI**
Cluster	1.29	0.28	0.83–1.98
**LR test vs. logistic regression:**	30.54, *p* < 0.0000		
**Residual mean (SD)**	3.32 * 10^−6^ (1.12 * 10^−6^)		
**Spearman's correlation coefficient**	0.850, *p* < 0.0001		
**Root MSE training dataset**	0.191, *p* = 0.63		
**Root MSE test dataset**	0.188, *p* = 0.72		

*Data are presented as adjusted Odds Ratio (OR_adj_), 95% confidence intervals (95% CI) and p-value*.

§ *Fenced is referred to all types of fences (double, single solid or metal fences)*.

The logistic mixed model results are reported in Table 5. The probability to observe a “*fast*” outbreak compared to “*normal*” outbreak was about 3 times more for farms located a distance <11.2 (mean value of the network) from the centroid of the cluster (OR_adj_ = 3.85, 95% CI = 2.05–7.20, *p* < 0.0001) and about 2 times if the farm was not fenced (OR_adj_ = 1.79, 95% CI = 1.65–1.97, *p* = 0.027). Increasing by one the number of days needed for outbreak confirmation and the number of symptomatic pigs, the probability of “*fast*” outbreak occurrence raised of 1% (OR_adj_ = 1.01, 95% CI = 1.01–1.02, *p* < 0.0001) and 7% (OR_adj_ = 1.07, 95% CI = 1.03–1.13, *p* = 0.001), respectively. Otherwise, the probability was about half when no one relationship with other farms was reported (OR_adj_ = 0.45, 95% CI = 0.22–0.76, *p* = 0.011), and reduced of about 60% when disposal clothing usage was declared (OR_adj_ = 0.63, 95% CI = 0.45–0.77, *p* < 0.0001). Border line higher probability (*p* = 0.045) was associated to those farms in which disinfection was not carried out (OR_adj_ = 1.05, 95% CI = 1.02–3.81). Considering the lower AIC value associated (AIC = 2254.33, delta AIC = 61.89), disinfection variable was included in the final model even if borderline. The predicted performance of the final models was tested by analyzing the regression's residuals, both within the “training dataset” (i.e., internal validation) and the “test dataset” (i.e., external validation). The models showed to be able to predict the correct outcome properly with a strong goodness-of-fit, according to internal and external validation criteria (residuals' mean, SD, Spearman's correlation coefficient). The root mean square tests were insignificant for both datasets indicating no evidence of failure.

**Table 5:** Logistic mixed model results using *fast* (1) or *normal* (0) outbreaks as outcome, with a time cut-off from primary case <20 days for categories definition, based on uniform-kernel-smoothed function.

**Outcome = fast outbreak detected by Uniform-kernel-smoothed function**			
**Variable**	**OR_**adj**_**	**95% CI**	***p***
**Distance < 11.2 km**	3.85	2.05–7.20	<0.0001
**Days for outbreak confirmation**	1.01	1.01–1.02	<0.0001
**No animals with symptoms**	1.07	1.03–1.13	0.001
**Disinfection**			
Yes Not	Ref. 1.05	1.02–3.81	0.045
**Type of fence**			
Fenced^§^ Not fenced	Ref. 1.79	1.65–1.97	0.027
**Relationship with other farms**			
Yes Not	Ref. 0.45	0.22–0.76	0.011
**Disposable clothing**			
Yes Not	Ref. 0.63	0.45–0.77	<0.0001
**Random effect**	**Est**	**SE**	**95% CI**
Year	0.63	0.25	0.22–1.54
Cluster	1.18	0.35	0.65–2.11
**LR test vs. logistic regression:**	9.49, *p* = 0.001		
**Residual mean (SD)**	1.52 * 10^−6^ (0.22 * 10^−6^)		
**Spearman's correlation coefficient**	0.790, *p* < 0.0001		
**Root MSE training dataset**	0.119, *p* = 0.32		
**Root MSE test dataset**	0.208, *p* = 0.55		

*Data are presented as odds ration adjusted (OR_adj_), 95% confidence intervals (95% CI) and p-value*.

§ *Fenced is referred to all types of fences (double, single solid or metal fences)*.

## Discussion

The present study examines 10 years of ASF outbreaks in domestic pig farms in Sardinia in depth and provides specific transmission network estimations for smallholder farms. Given their often low biosecurity level, smallholder farms are considered particularly susceptible to ASFV introduction and are of particular interest in disease prevention and control (36). In addition, even though small-scale farming represents a fundamental part of agricultural practices and is common in rural areas (67), biosecurity and management practices have been described mainly for intensive pig farms (68–71) and focus on backyard farms in non-European countries (35, 72–74). Due to the spread of ASF in European countries with a relevant backyard pig production, it is likely that this issue is even more widespread (32). A pioneering European study focusing on smallholder traditional pig management practices was carried out in Corsica in 2015 and quantified the risk associated with free-range breeding, improper storage of carcasses and distribution of kitchen waste in pastures (75). More recently, in Romania, most of the outbreaks have been significantly associated with the immediate context (<2 km) of ASFV circulation (i.e., increasing number of outbreaks in domestic farms and wild boar around these farms). Importantly, the same study associated the risk of ASF introduction in backyard farms with the herd size, visits by professionals working on farms and pigs foraging in ASF-affected areas (33). Most of these studies recognize humans as being mainly responsible for both long-distance transmission and virus introduction in domestic pig farms, which are mostly comprised of small-scale pig holdings in rural areas (34–36). All of these studies underline the need for awareness-raising campaigns among all stakeholders to sensitize farmers to proper biosecurity practices and the provision of incentives for farmers to report suspected outbreaks to authorities for rapid confirmation (30–35, 72–75). Furthermore, all these studies underlined the need of take into account the context when dealing with non-commercial holdings, in order to ensure survival of these traditional farming methods that express the cultural identity of many countries (42, 74–76). However, despite the fact that these studies provide risk factor estimation, they lack comparisons between intensive and small-scale holdings and a measure of disease spread. To the best of our knowledge, this is the first study able to investigate smallholders' practices concerning biosecurity measures in European countries, and to provide details and estimation of the target smallholder farms where the virus could spread more faster. Furthermore, this study highlight the need of detailed epidemiological farm investigations, including the tracing of contact farms to identify sources of infection, essential for early detection and stop the virus spread (77). ASFV has remained in circulation in Sardinia for more than 40 years, and even though the last PCR-positive detection dates back to 2019 in wild boar, the island still remains categorized amongst the highest risk areas in the EU, according to the newly adopted Commission Implementing Regulation n. 2021/605, with consequent very severe trade restrictions still already in place. Given the strong correlation between the number of *fast* outbreaks and the number of secondary outbreaks in each cluster, characterizing the features associated with the *fast* outbreaks is of great concern to risk evaluation. These fast outbreaks tend to spread to more farms, and thus complicate control efforts and increase costs to both farmers and authorities. Some of the key features associated with faster virus spread highlighted in this study (i.e., outdoor farms, familiar or working relationship with other farms, low-distance, number of animals breed, absence of adequate disinfection) are common risk factors identified in previous studies focusing on smallholder pig farms (32–36, 42, 43, 73–78). The baseline ASF risk of outdoor farms identified in this study (15–20%) well-reflects the last estimation published by EFSA in an hypothetical scenario where no outdoor-specific biosecurity measures are implemented (31). Even if the number of secondary cases within the cluster was similar if the primary case was indoor or outdoor, the nearest-neighbor analysis underlined the higher probability of outdoor to be untimely infected by ASFV rather than indoor farms, suggesting the central role of direct contact between animals in space-limited clusters, particularly in farms where the animals have access to yard or runs. Furthermore, applying the kernel transmission functions, measures of association and risk estimation that have never been published before were provided, highlighting the low probability of fast outbreak if the farm is adequately fenced, the importance of hygiene and disinfection in preventing the speed of disease transmission and the key role of farmers who hunt wild boar. Indeed, the analysis show the clear effect of the last control measures implemented against free-ranging pigs (39) on the decreasing incidence of outbreaks in Sardinia. The results underline the central role of free-ranging pigs as population link between domestic and wild population. Furthermore, the mean transmission distance estimation suggests the key role of farmers and, more generally, of the human population, in the spread of ASFV in Sardinia. However, this study highlighted a flaw in the surveillance system before 2016: undetected outbreaks with associated spreading of the disease throughout the infected zone and possible unreported cases were not considered in this study. The comparison between the possible transmission network described by epidemiological investigations and those generated by kernels functions highlights the substantial agreement of this tool in estimating epidemiological correlation between near outbreaks, but its moderate agreement in matching long-distance events. Otherwise, statistical models are unable to predict unlikely events, such as ASFV transmission by frozen meat over 70 days without virus detection. Veterinarians on field experience may be more efficient in this regards. The number of secondary cases estimated by both the kernel functions confirm the period between April and June as the at most risk period for ASFV transmission, as previously underlined by the same authors (40). Furthermore, the data source represented by the epidemiological investigations could have generated some reporting errors, affecting, at least partially, the robustness of the study as well as the possible reporting delay by veterinarian authorities. The recent systematic review carried out by Hayes et al. (79) empathizes the need of take into account the epidemiological context, particularly incorporating ASF transmission between pigs and boar in transmission models (79). We tried to cover at least partially this gap implementing the kernel functions with the wild boar density which play a strategic role in ASFV transmission and disease endemicity (6, 39). Finally, the parameters estimated have to be carefully evaluated before generalization, given the particular Sardinian context, not only for the presence of three suid populations typical of the island (6, 39, 40), but also for the types of domestic pig farms that are mainly intended for self-consumption. Otherwise, the applied methods and the obtained results could be efficiently applied in other contexts where outdoor farming system is a traditional farming methods, or in EU countries close to eradication. Considering the partial identification of outdoor farms as the target population for the ASFV, the author strictly agree with the need of specific support (i.e., economic, veterinary services) for smallholders to ensure survival of these traditional farm and not to put them at a disadvantage (75, 77). Indeed, the feasibility and sustainability of specific control measures such as double fence and not outdoor access must be evaluated in each context to encourage ongoing improvement of on-farm biosecurity (31), avoiding stronger measures inapplicable, which could likely generate farmer disagreement or even more illegality (78). Otherwise, identify the most at risk period and the target farm population is essential to put in place efficient control measures.

## Conclusion

The main conclusions that can be drawn from our results on ASF occurrence in pig farms in Sardinia are as follows:

(1) Faster spread of the disease was influenced by the type of farm, distance between them, management and epidemiological context;
(2) Considering the number of secondary cases estimated, this study underline the importance of the epidemiological investigation report and the need of improve this tool, in order to speed up its ability in detecting long-distance epidemiological correlations;
(3) The detection system has not always led to early virus detection in relation to secondary outbreaks, thus the sensitivity of the early detection system needs to be estimated and the system adjusted accordingly;
(4) The measures recommended to obtain high biosecurity levels should be flexible and should take into account local conditions;
(5) The results of this study confirm that the overall measures adopted to eradicate ASF in Sardinia in the last years have had a major favorable impact on disease occurrence in pig farms.

## Data Availability Statement

The original contributions presented in the study are included in the article/Supplementary Material, further inquiries can be directed to the corresponding author.

## Author Contributions

SR, FL, and SC: conceptualization. FL and VG: methodology. DM and FL: software and validation. FL: formal analysis, resources, project administration, and funding acquisition. DM: investigation and data curation. FL and SC: writing—original draft preparation and visualization. SR, DM, FL, AO, SD, GF, VG, and SC: writing—review and editing. SR, AO, VG, and SC: supervision. All authors have read and agreed to the published version of the manuscript.

## Conflict of Interest

The authors declare that the research was conducted in the absence of any commercial or financial relationships that could be construed as a potential conflict of interest.

## Publisher's Note

All claims expressed in this article are solely those of the authors and do not necessarily represent those of their affiliated organizations, or those of the publisher, the editors and the reviewers. Any product that may be evaluated in this article, or claim that may be made by its manufacturer, is not guaranteed or endorsed by the publisher.

## Supplementary Material

Supplementary Figure 1 Exponentially increasing intensity of secondary cases with increasing wild boar population density values (wild boar/km2), when the population density is expressed as a log. The colored envelopes represent the 95% confidence intervals.

Supplementary Table 1 List of the variable collected by the Epidemiological Investigation tools.
