# Supplementary figures and images for: African Swine Fever in Smallholder Sardinian Farms: Last 10 Years of Network Transmission Reconstruction and Analysis
Source: Front Vet Sci. 2021 Jul 30;8:692448. doi: 10.3389/fvets.2021.692448 (PMC8361751; doi:10.3389/fvets.2021.692448)

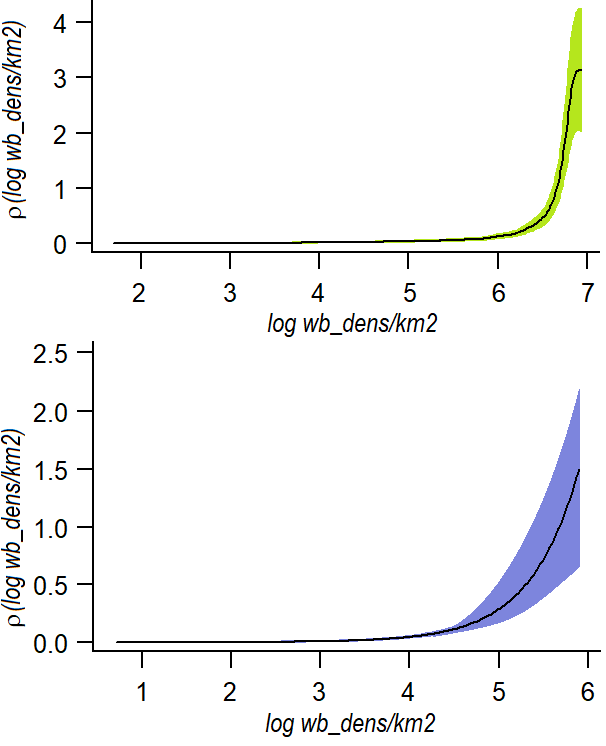

Supplement: Supplementary Figure 1 — Exponentially increasing intensity of secondary cases with increasing wild boar population density values (wild boar/km2), when the population density is expressed as a log. The colored envelopes represent the 95% confidence intervals. [file Image_1.TIF]
