# Supplementary material for: African Swine Fever in Smallholder Sardinian Farms: Last 10 Years of Network Transmission Reconstruction and Analysis
Source: Front Vet Sci. 2021 Jul 30;8:692448. doi: 10.3389/fvets.2021.692448 (PMC8361751; doi:10.3389/fvets.2021.692448)
Supplement: Supplementary Table 1 — List of the variable collected by the Epidemiological Investigation tools. [file Table_1.DOCX]

# Supplementary Material

**Table S1**. List of the variable collected by the Epidemiological Investigation tools.

| **Variables** | Type of variable |
| --- | --- |
| Farm code | Number |
| Data of epidemiological investigation | From 2010 to 2018 |
| Data of ASF outbreak | From 2010 to 2018 |
| Municipality | 87 over 377 Sardinian municipalities |
| Province | All 5 Sardinian provinces |
| Location | lat, long |
| Production type | Close or open cycle, self-consumption |
| Animal management | Fenced outdoor, indoor |
| Data last veterinary control | < 6 months, 6 months-1 year, >1 year |
| Total N. of animals | N umber |
| N. died animals | Number (%) |
| N. animals with symptoms | Number (%) |
| Type of symptoms | Descriptive |
| Data of symptoms appearance | dd/mm/yyyy |
| Data of suspicion | dd/mm/yyyy |
| Data of virus isolations | dd/mm/yyyy |
| Type of fence | Single solid fence, double fence, single metal net, not fenced |
| Estimated occasional contact with wild boar | Dichotomous, yes or not |
| Minimum distance with other farms | Kilometers (km) |
| Declared relationship with other farms | Family relationship, working collaboration, other |
| Farmer as hunter | Dichotomous, yes or not |
| Presence of slaughterhouse in farm | Dichotomous, yes or not |
| Shelter | Open, close |
| Loading and unloading | Inside farm, outside farm |
| Quarantine | Dichotomous, yes or not |
| Animal identification | Dichotomous, yes or not |
| Farm register compiled | Dichotomous, yes or not |
| Disinfection | Dichotomous, yes or not |
| Disposable clothing | Dichotomous, yes or not |
| Animal separation by categories | Dichotomous, yes or not |
| Storage of livestock waste/manure | Dichotomous, yes or not |
| Closed carcasses storage | Dichotomous, yes or not |
| Pigs biting with kitchen waste | Dichotomous, yes or not |
| Epidemiologically correlated with other farms | Dichotomous, yes or not |
| Farm code epidemiologically correlated | Number |
| Origin of contagious | Descriptive |
